# Differential synovial tissue biomarkers among psoriatic arthritis and rheumatoid factor/anti-citrulline antibody-negative rheumatoid arthritis

**DOI:** 10.1186/s13075-019-1898-7

**Published:** 2019-05-09

**Authors:** Stefano Alivernini, Dario Bruno, Barbara Tolusso, Laura Bui, Luca Petricca, Maria Rita Gigante, Domenico Birra, Anna Laura Fedele, Giusy Peluso, Francesco Federico, Gianfranco Ferraccioli, Elisa Gremese

**Affiliations:** 1 Division of Rheumatology, Fondazione Policlinico Universitario A. Gemelli IRCCS, Rome, Italy; 2 Institute of Rheumatology, Università Cattolica del Sacro Cuore, Rome, Italy; 3 Institute of Pathology, Fondazione Policlinico Universitario A. Gemelli IRCCS, Rome, Italy; 4 Institute of Pathology, Università Cattolica del Sacro Cuore, Rome, Italy

**Keywords:** Psoriatic arthritis, Rheumatoid arthritis, Synovial tissue, Autoantibodies, Response to therapy

## Abstract

**Background:**

Differential diagnosis among psoriatic arthritis (PsA) and seronegative rheumatoid arthritis (Ab^neg^ RA) can be challenging particularly in the clinical setting of peripheral phenotype and autoantibodies seronegativity. The aim of the study was to identify synovial tissue (ST) biomarkers differentially expressed in PsA and Ab^neg^ RA and test their predictive value of therapeutic response.

**Methods:**

Thirty-four PsA patients [12 DMARD naive and 22 non-responder to methotrexate (MTX-IR)] with peripheral joint involvement and 55 Ab^neg^ RA (27 DMARD naive and 28 MTX-IR) underwent US-guided ST biopsy and immunohistochemistry (IHC) for CD68^+^, CD3^+^, CD20^+^, CD21^+^, CD117^+^, and CD138^+^ cells. After study entry, each DMARD-naive patient started MTX therapy and was followed in an outpatient setting for at least 6 months to define the achievement of Minimal Disease Activity (PsA) and DAS remission (Ab^neg^ RA) status respectively. Each IR-MTX patient was treated according to EULAR recommendations.

**Results:**

At study entry, IHC analysis revealed that PsA patients had comparable levels of lining and sublining CD68^+^ and sublining CD21^+^, CD20^+^, and CD3^+^ cells than Ab^neg^ RA, despite the therapeutic regimen. Moreover, regardless of the therapeutic scheme, PsA patients showed higher IHC score of CD117^+^ cells (*p* = 0.0004 and *p* = 0.0005 for naive and MTX-IR patients respectively) compared to Ab^neg^ RA patients. Conversely, Ab^neg^ RA patients showed higher IHC score of CD138^+^ cells, irrespective to the therapeutic scheme (*p* = 0.04 and *p* = 0.002 for naive and MTX-IR patients respectively). Analyzing the response rate to the therapeutic scheme, naive PsA patients reaching MDA status at 6 months follow-up, showed, at the study entry, lower IHC score of CD3^+^ cells compared to PsA patients not reaching this outcome (*p* = 0.02); conversely, naive Ab^neg^ RA patients reaching DAS remission status at 6 months follow-up, showed, at the study entry, lower IHC score of sublining CD68^+^ cells compared to Ab^neg^ RA patients not reaching this outcome (*p* < 0.001).

**Conclusions:**

CD117^+^ and CD138^+^ cells are differentially distributed among PsA and Ab^neg^ RA. Histological analysis of ST may help to solve the clinical overlap between the two diseases and provides prognostic data about the therapy success.

**Electronic supplementary material:**

The online version of this article (10.1186/s13075-019-1898-7) contains supplementary material, which is available to authorized users.

## Introduction

Psoriatic arthritis (PsA) is a chronic inflammatory disease characterized by remarkable heterogeneity of clinical presentation, including peripheral arthritis, axial involvement, enthesitis, dactylitis, nail dystrophy, uveitis, and osteitis, in addition to associated comorbidities such as cardiovascular disease, metabolic syndrome, and mood disorders [1, 2]. Most patients with PsA present with oligoarticular or polyarticular arthritis and can be differentiated from patients with rheumatoid arthritis (RA), the most common inflammatory joint disease, by specific non-articular clinical features being present, as well as the infrequent seropositivity for rheumatoid factor (RF) and anti-citrullinated peptide antibody (ACPA). These clinical features include an asymmetric distribution of the inflamed joints, the sacroiliitis or spinal involvement, the typical involvement of the distal interphalangeal joint (DIP), and the extra-articular manifestations [1]. However, in clinical practice, the differential diagnosis among PsA and RA can be challenging, particularly if the peripheral phenotype is present and RF and ACPA are negative. In recent years, there have been a number of advances made in synovial tissue biopsy techniques in patients with inflammatory joint diseases [3]. However, despite many research groups have focused on the analysis of possible differential biomarkers among PsA and RA, no studies have been performed considering a direct comparison between PsA with a RF/ACPA seronegative (Ab^neg^) RA cohort [4, 5].

Based on that, the aims of the study were (i) to assess whether the histological characteristics of synovial tissue of PsA patients with peripheral arthritis phenotype compared to seronegative RA patients could differ in different disease phases and (ii) to evaluate possible predictive synovial biomarkers associated with treatment response in PsA and seronegative RA patients at disease onset and after c-DMARD failure.

## Patients and methods

### Patient enrollment

Eighty-nine patients were enrolled in the study [34 oligo-polyarticular PsA patients (12 naïve and 22 inadequately responder to methotrexate (MTX-IR) respectively) and 55 RA patients (27 naïve and 28 MTX-IR respectively)] fulfilling the classification criteria for PsA or RA [6, 7]. At baseline, demographical, clinical, and inflammatory parameters were collected for each patient. All RA patients were confirmed as being seronegative (Ab^neg^) for IgA- and IgM-RF (Orgentec Diagnostika, Bouty, UK) and ACPA (Axis Shield Diagnostics, Bouty, UK) using commercial ELISA at study entry. Each naïve PsA and Ab^neg^ RA patient was then treated with methotrexate (up to 20 mg/week) and followed every 3 months for at least 6 months to assess the rate of achievement of Minimal Disease Activity (MDA) or DAS remission for PsA and Ab^neg^ RA patients respectively [8, 9]. MTX-IR PsA and Ab^neg^ RA patients were treated according to the current recommendations [10, 11]. The study protocol was approved by the local Ethical Committee, and all subjects provided signed informed consent.

### Immunohistochemistry for CD68, CD21, CD3, CD20, CD138, CD117, and CD31 on synovial tissue

At study entry, each PsA and Ab^neg^ RA patient underwent ultrasound-guided synovial tissue biopsy of the knee. Joint inflammatory activity was tested for each patient using power Doppler as previously described [12]. Each tissue was tested through immunohistochemistry for the presence of CD68 (macrophages), CD21 (follicular dendritic cells), CD3 (T lymphocytes), CD20 (B lymphocytes), and CD31 (endothelial cells) staining following the already published protocol [12]. Other tissue sections were stained for plasma cell and mast cell detection, using CD138 mouse antihuman monoclonal antibody (clone MI15) or CD117 mouse antihuman monoclonal antibody (clone EP10) (all from Leica Biosystem, Newcastle, UK) by immunostainer BOND MAX III (Leica). Slides were examined by two independent evaluators using a light microscope (Leica DM 2000), and all tissues were evaluated using a numerical score based on the number of CD68^+^, CD21^+^, CD3^+^, CD20^+^, CD117^+^, and CD138^+^ cells (two different fields in each section), with a score of 0 indicating no positive cells, 1 indicating < 10% positive cells, 2 indicating 10–50% positive cells, and 3 indicating > 50% positive cells. CD31^+^ vessel count was done as mean of the values from three different fields in each section [12]. The inter-rater agreement coefficient was assessed for each single IHC marker (see Additional file 2: Table S1).

### Statistical analysis

Statistical analysis was performed using SPSS V. 20.0 (SPSS. Chicago, IL, USA) and Prism software (GraphPad, San Diego, CA, USA). Categorical and quantitative variables were described as frequencies, percentage, and mean ± SD. Data on demographic and clinical features were compared between patients by the non-parametric Mann-Whitney *U* test or *χ*^2^ test, as appropriate. Spearman’s rank correlation test was used for correlation in all analyses. ROC analysis was performed to identify the best cut-off value for IHC scores associated with the highest rate of MDA or DAS remission achievement in PsA and Ab^neg^ RA patients respectively. A value of *p* ≤ 0.05 was considered statistically significant.

## Results

### Demographic and clinical characteristics of the enrolled study cohorts

Demographic and clinical characteristics of PsA and Ab^neg^ RA cohorts (naïve and MTX-IR respectively) enrolled in the study are summarized in Table 1. Comparing the different study cohorts, there were no significant differences according to age and gender (*p* > 0.05). PsA and Ab^neg^ RA patients naïve to treatment showed significant shorter disease duration (0.80 ± 0.24 years for naive PsA and 0.84 ± 0.95 Ab^neg^ RA patients respectively) compared to MTX-IR PsA and Ab^neg^ RA patients (5.83 ± 3.68 years for MTX-IR PsA, *p* < 0.001; 5.68 ± 5.28 for MTX-IR Ab^neg^ RA patients, *p* = 0.002 respectively). Considering the clinical parameters, there were no significant differences comparing PsA and Ab^neg^ RA patients based on swollen and tender joint counts or Disease Activity Scores in naïve and in MTX-IR subgroups (Table 1). However, considering inflammatory markers, ESR plasma levels were significantly higher in naïve Ab^neg^ RA patients (44.00 ± 25.40 mm/first hour) than MTX-IR Ab^neg^ RA patients (29.60 ± 36.94 mm/first hour; *p* = 0.03), whereas no differences were found for CRP plasma levels comparing naïve and MTX-IR PsA subgroups (Table 1).

**Table 1:** Demographical, clinical, and immunological characteristics of the study cohorts

	PsA (*n* = 34)		*p*	Ab^neg^ RA (*n* = 55)		*p* ^1^	*p* ^2^	*p* ^3^
	Naive (*n* = 12)	MTX-IR (*n* = 22)		Naive (*n* = 27)	MTX-IR (*n* = 28)			
Age, years (mean ± SD)	54.75 ± 17.18	60.18 ± 9.81	0.47	52.41 ± 17.70	58.36 ± 16.58	0.16	0.68	0.77
Female, *n* (%)	4 (33.3)	14 (63.6)	0.10	23 (85.2)	23 (82.1)	0.76	*0.001*	0.14
Disease duration, years (mean ± SD)	0.80 ± 0.24	5.83 ± 3.68	*< 0.001*	0.84 ± 0.95	5.68 ± 5.28	*0.002*	0.44	0.49
DAS44 (mean ± SD)	3.00 ± 0.42	3.34 ± 0.91	0.34	3.37 ± 1.05	3.22 ± 0.79	0.71	0.52	0.81
DAPSA (mean ± SD)	22.55 ± 8.38	28.04 ± 10.14	0.10	–	–	–	–	–
SJC (mean ± SD)	4.00 ± 2.67	6.43 ± 5.69	0.35	8.22 ± 7.95	7.15 ± 5.53	0.93	0.18	0.54
TJC (mean ± SD)	5.00 ± 3.16	7.00 ± 6.13	0.66	8.22 ± 7.86	8.44 ± 5.46	0.33	0.51	0.24
ESR, mm/1st hour (mean ± SD)	29.60 ± 36.94	29.10 ± 19.59	0.44	44.00 ± 25.40	37.04 ± 27.25	0.19	*0.03*	0.44
CRP, mg/l (mean ± SD)	16.70 ± 29.16	18.11 ± 23.41	0.70	16.99 ± 19.43	16.77 ± 24.23	0.77	0.72	1.00
Ab positivity, *n* (%)	0 (0.0)	0 (0.0)	1.00	0 (0.0)	0 (0.0)	1.00	1.00	1.00

Data presented in italics have *p* < 0.05

*PsA* psoriatic arthritis, *RA* rheumatoid arthritis, *Ab* autoantibody, *DAS* Disease Activity Score, *DAPSA* Disease Activity in PSoriatic Arthritis, *SJC* swollen joint count, *TJC* tender joint count, *ESR* erythrocyte sedimentation rate, *CRP* C-reactive protein, *SD* standard deviation, *MTX-IR* methotrexate inadequately responder, *p* naive PsA vs MTX-IR PsA, *p*^*1*^ naive Ab^neg^ RA vs MTX-IR Ab^neg^ RA, *p*^*2*^ naive PsA vs naive Ab^neg^ RA, *p*^*3*^ MTX-IR PsA vs MTX-IR Ab^neg^ RA

### PsA and Ab^neg^ RA show similar histological features in terms of synovial resident CD68^+^, CD21^+^, and CD3^+^ cells and microanatomical organization

**Fig. 1:**
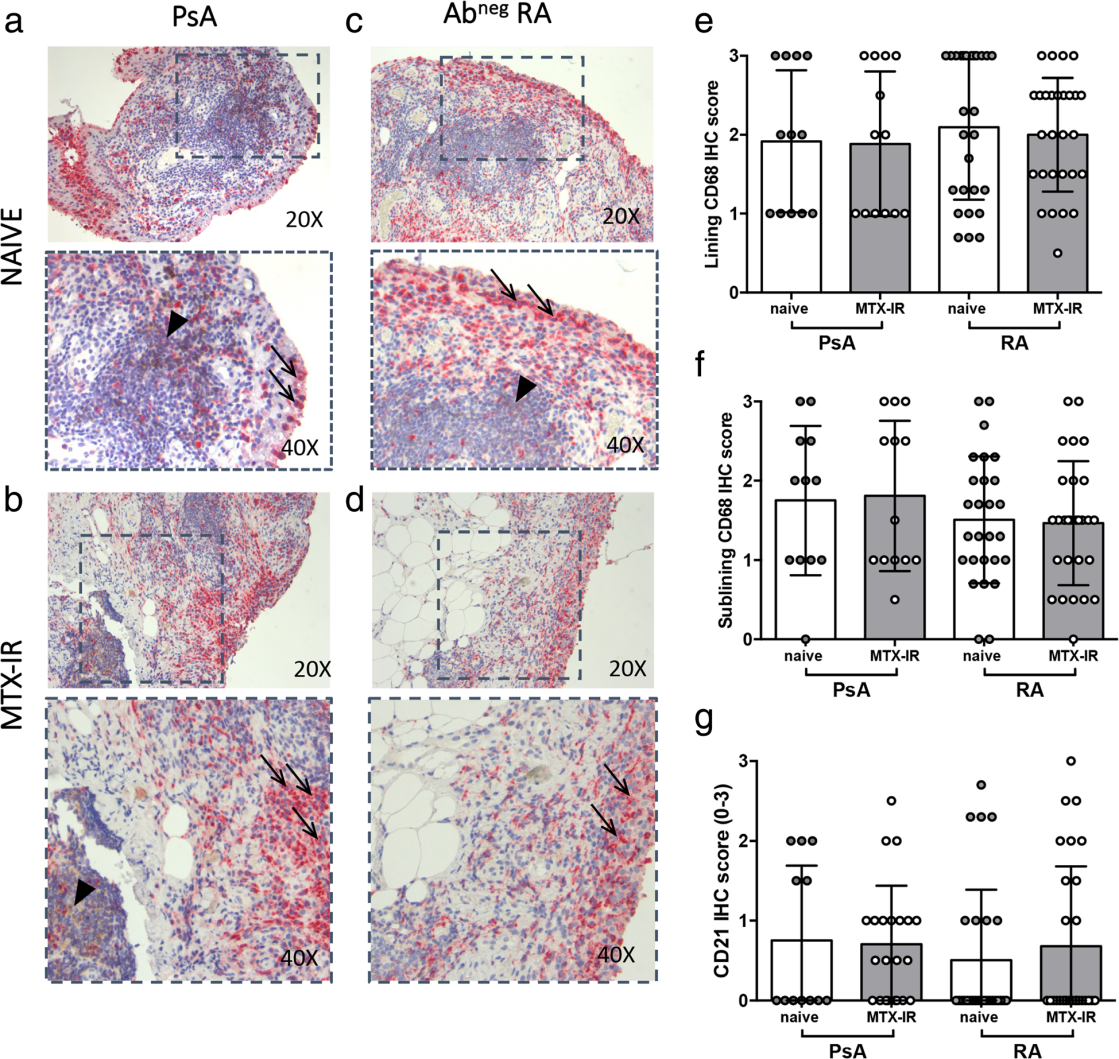
**a**–**g** IHC staining for CD68/CD21 on ST of patients with naïve or MTX-IR PsA and Ab^neg^ RA patients. Example photos of CD68 (RED)/CD21(DAB) staining of ST biopsies from patients with naïve (**a**) or MTX-IR (**b**) PsA patients and with naïve (**c**) or MTX-IR (**d**) Ab^neg^ RA (magnification × 20 and magnification × 40 in the corresponding inset). Black arrows indicate CD68^+^ cells (red), and black arrow head indicates CD21^+^ cells (brown) in the corresponding inset. **e** Lining IHC score for CD68^+^ cells in PsA and Ab^neg^ RA patients divided by treatment regimen. **f** Sublining IHC score for CD68^+^ cells in PsA and Ab^neg^ RA patients divided by treatment regimen. **g** IHC score for CD21^+^ cells in PsA and Ab^neg^ RA patients divided by treatment regimen. IHC immunohistochemistry, PsA psoriatic arthritis, RA rheumatoid arthritis, Ab autoantibody, MTX-IR methotrexate inadequately responder, CD cluster designation

Each enrolled patient underwent US-guided ST biopsy, and IHC for CD68^+^, CD21^+^, CD20^+^, and CD3^**+**^ was performed. PsA patients showed similar IHC CD68^+^ cell score in the lining (1.95 ± 0.90) and sublining (1.81 ± 0.95) compared to Ab^neg^ RA patients (1.96 ± 0.75 for lining CD68^+^ cells, *p* = 0.94; 1.43 ± 0.76 for sublining CD68^+^ cells, *p* = 0.11 respectively) regardless of the treatment scheme (Fig. 1a–d). Furthermore, IHC analysis revealed that PsA and Ab^neg^ RA patients had comparable levels of CD21^+^ cells (0.72 ± 0.79 vs 0.60 ± 0.95; *p* = 0.18 respectively) regardless of the treatment scheme (Fig. 1a–d).

Stratifying the study cohorts according to the treatment scheme, naïve PsA showed similar IHC scores for lining (2.00 ± 0.85) and sublining CD68^+^ cells (1.71 ± 0.96) compared to naïve Ab^neg^ RA patients (1.96 ± 0.79 for lining CD68^+^ cells, *p* = 1.00; 1.44 ± 0.80 for sublining CD68^+^ cells, *p* = 0.39 respectively). Moreover, naïve PsA showed similar IHC scores for CD21^+^ cells (0.75 ± 0.94) compared to naïve Ab^neg^ RA patients (0.52 ± 0.91; *p* = 0.43) (Fig. 1g), showing a direct correlation between ESR plasma levels and lining CD68^+^ cells IHC score (*r* = 0.66; *p* = 0.04). Similarly, MTX-IR PsA patients showed comparable IHC scores for lining (1.92 ± 0.95) and sublining CD68^+^ cells (1.86 ± 0.96) than MTX-IR Ab^neg^ RA patients (2.00 ± 0.72 for lining CD68^+^ cells, *p* = 0.79; 1.46 ± 0.78 for sublining CD68^+^ cells, *p* = 0.25) (Fig. 1e–h). Finally, MTX-IR PsA showed similar IHC scores for CD21^+^ cells (0.71 ± 0.73) compared to MTX-IR Ab^neg^ RA patients (0.68 ± 1.00; *p* = 0.91) (Fig. 1g).

Analyzing the microanatomical organization of the synovial tissue infiltrates, 15 (44.1%) PsA patients compared to 24 (43.6%, *p* = 0.51) Ab^neg^ RA patients showed follicular synovitis regardless of the treatment regimen. Moreover, there was no difference in the rate of follicular synovitis stratifying patients according to the therapeutic regimen (41.7% of naïve PsA patients with follicular synovitis compared to 45.5% of MTX-IR PsA patients with similar synovitis pattern, *p* = 0.79; 44.4% of naïve Ab^neg^ RA patients with follicular synovitis compared to 42.8% of MTX-IR Ab^neg^ RA patients with similar synovitis pattern, *p* = 0.51).

**Fig. 2:**
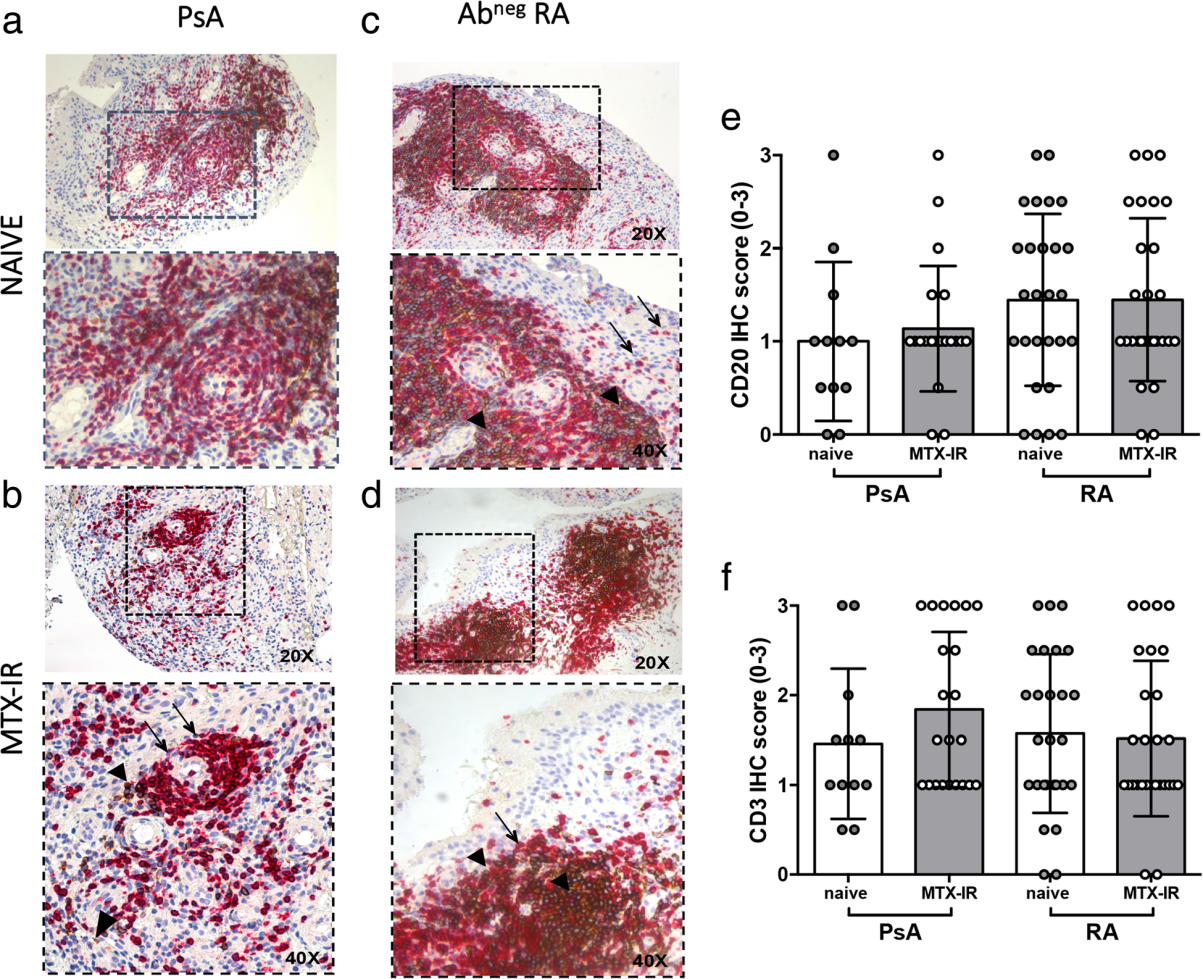
**a**–**f** IHC staining for CD3/CD20 on ST of patients with naïve or MTX-IR PsA and Ab^neg^ RA patients. Example photos of CD3 (RED)/CD20 (DAB) staining of ST biopsies from patients with naïve (**a**) or MTX-IR (**b**) PsA patients and with naïve (**c**) or MTX-IR (**d**) Ab^neg^ RA patients (magnification × 20 and magnification × 40 in the corresponding inset). Thin black arrows indicate CD3^+^ cells (red), and black arrow heads indicate CD20^+^ cells (brown) in the corresponding inset. **e** IHC scores for CD20^+^ cells in PsA and Ab^neg^ RA patients divided by treatment regimen. **f** IHC scores for CD3^+^ cells in PsA and Ab^neg^ RA patients divided by treatment regimen. IHC immunohistochemistry, PsA psoriatic arthritis, RA rheumatoid arthritis, Ab autoantibody, MTX-IR methotrexate inadequately responder, CD cluster designation

CD20 IHC revealed that PsA and Ab^neg^ RA patients had similar levels of CD20^+^ cells (1.08 ± 0.73 in PsA vs 1.44 ± 0.89 in Ab^neg^ RA, *p* = 0.07) (Fig. 2a–d) and after stratification based on the treatment regimen, PsA patients showed similar IHC CD20^+^ cell score than Ab^neg^ RA (1.00 ± 0.85 in naive PsA vs 1.44 ± 0.92 in naive Ab^neg^ RA, *p* = 0.16; 1.14 ± 0.67 in MTX-IR PsA vs 1.45 ± 0.87 MTX-IR Ab^neg^ RA, *p* = 0.18) (Fig. 2e).

Analyzing synovial CD3^+^ cell distribution, IHC showed that PsA and Ab^neg^ RA patients had similar levels of synovial CD3^+^ cells (1.71 ± 0.86 in PsA and 1.54 ± 0.86 in Ab^neg^ RA; *p* = 0.45 respectively) (Fig. 2a–d). Moreover, PsA and Ab^neg^ RA patients did not differ in terms of synovial CD3^+^ cells stratifying patients based on the treatment scheme (1.45 ± 0.83 in naive PsA vs 1.57 ± 0.88 in naïve Ab^neg^ RA, *p* = 0.70; 1.84 ± 0.86 in MTX-IR PsA vs 1.52 ± 0.87 MTX-IR Ab^neg^ RA, *p* = 0.19) (Fig. 2f).

### Synovial CD117^+^ and CD138^+^ cells are differentially distributed in PsA and Ab^neg^ RA patients

**Fig. 3:**
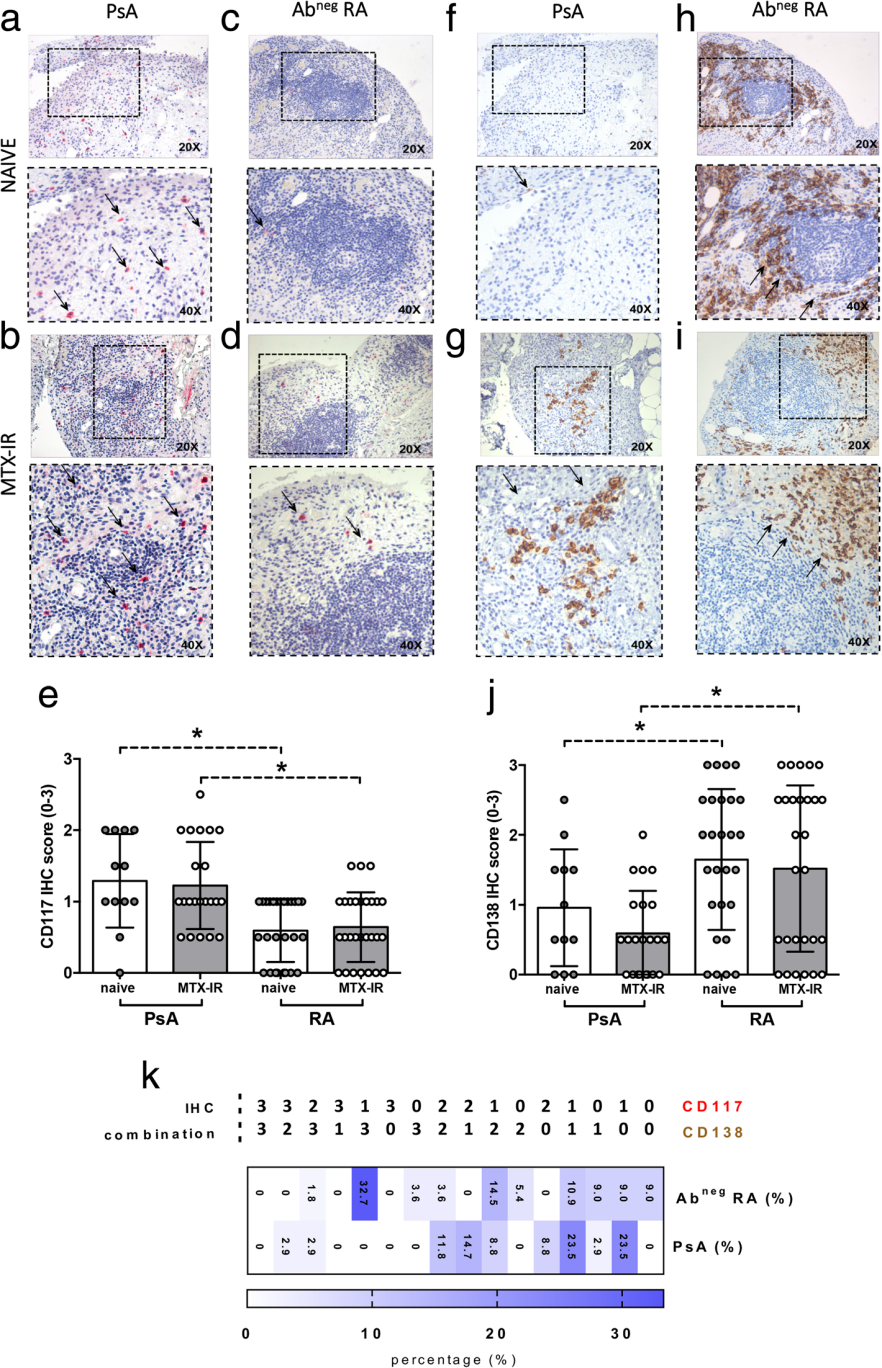
**a**–**k** IHC staining for CD117 and CD138 on ST of patients with naïve or MTX-IR PsA and Ab^neg^ RA patients. Example photos of CD117 (RED) staining of ST biopsies from patients with naïve (**a**) or MTX-IR (**b**) PsA and with naïve (**c**) or MTX-IR (**d**) Ab^neg^ RA (magnification × 20 and magnification × 40 in the corresponding inset). Thin black arrows indicate CD117^+^ cells (red) in the corresponding inset. **e** IHC scores for CD117^+^ cells in PsA and Ab^neg^ RA patients divided by treatment regimen; *p = 0.0004, naïve PsA vs naïve Ab^neg^ RA patients; *p = 0.0005, MTX-IR PsA vs MTX-IR Ab^neg^ RA patients. Example photos of CD138 (DAB) staining of ST biopsies from patients with naïve (**f**) or MTX-IR (**g**) PsA and with naïve (**h**) or MTX-IR (**i**) Ab^neg^ RA (magnification × 20 and magnification × 40 in the corresponding inset). Thin black arrows indicate CD138^+^ cells (brown) in the corresponding inset. **j** IHC scores for CD138^+^ cells in PsA and Ab^neg^ RA patients divided by treatment regimen; *p = 0.04, naive PsA vs naive Ab^neg^RA patients; *p = 0.002, MTX-IR PsA vs MTX-IR Ab^neg^ RA patients. **k** Rate of distribution of CD117/CD138 IHC combination differentially distributed among PsA and Ab^neg^ RA patients. IHC immunohistochemistry, PsA psoriatic arthritis, RA rheumatoid arthritis, Ab autoantibody, MTX-IR methotrexate inadequately responder, CD cluster designation

CD117 IHC showed that PsA patients are characterized by higher IHC scores for CD117^+^ cells (1.25 ± 0.61) compared to Ab^neg^ RA patients (0.62 ± 0.46, *p* < 0.001) regardless of the treatment scheme (Fig. 3a–d). Interestingly, stratifying the study cohorts according to the treatment scheme, naïve PsA patients showed higher CD117^+^ cell IHC scores (1.29 ± 0.65) compared to naïve Ab^neg^ RA patients (0.59 ± 0.44; *p* = 0.0004). Similarly, MTX-IR PsA patients showed higher CD117^+^ IHC score (1.23 ± 0.61) compared to MTX-IR Ab^neg^ RA patients (0.64 ± 0.48, *p* = 0.001) (Fig. 3e).

Conversely, analyzing the distribution of CD138^+^ cells within the synovial tissue, Ab^neg^ RA patients showed higher IHC scores for CD138^+^ cells (1.58 ± 1.09) compared to PsA patients (0.72 ± 0.71; *p* < 0.001) independently of the treatment scheme (Fig. 3f–i). Considering the different study population subgroups, naive Ab^neg^ RA patients were characterized by higher CD138^+^ cell IHC score (1.65 ± 1.00) compared to naive PsA patients (0.96 ± 0.84; *p* = 0.04). Similarly, MTX-IR Ab^neg^ RA patients had higher CD138^+^ cell IHC score (1.51 ± 1.19) compared to MTX-IR PsA patients (0.59 ± 0.61, *p* = 0.002) (Fig. 3j). As shown in Fig. 3k, the combination of low IHC score for CD117^+^ (IHC score = 1) and high IHC score for CD138^+^ cells (IHC score = 3) significantly differentiates PsA than Ab^neg^ RA synovitis [OR (95% CI), 34.04 (1.535–2.398); *p* = 0.0002].

### Synovial CD31^+^ vessel count is associated with the disease phase in PsA and Ab^neg^ RA patients

**Fig. 4:**
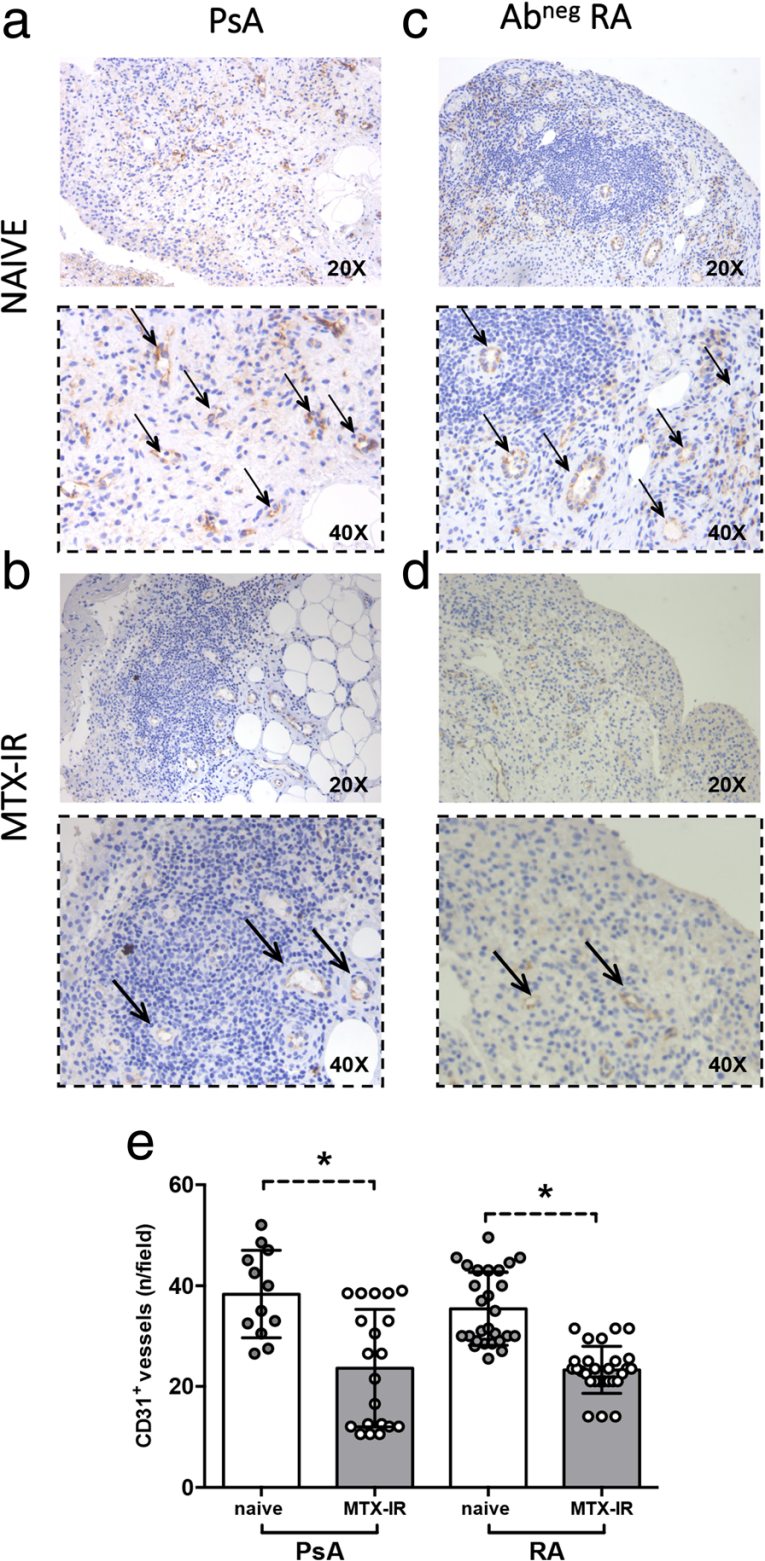
**a**–**e** IHC staining for CD31 on ST of patients with naïve or MTX-IR PsA and Ab^neg^ RA patients. Example photos of CD31 (DAB) staining of ST biopsies from patients with naïve (**a**) or MTX-IR (**b**) PsA and with naïve (**c**) or MTX-IR (**d**) Ab^neg^ RA (magnification × 20 and magnification × 40 in the corresponding inset). Thin black arrows indicate CD31^+^ vessels (brown) in the corresponding inset. **e** Synovial CD31^+^ vessel count in PsA and Ab^neg^ RA patients divided by treatment regimen; *p = 0.01, naïve vs MTX-IR PsA patients; *p < 0.001, naïve vs MTX-IR Ab^neg^ RA patients. IHC immunohistochemistry, PsA psoriatic arthritis, RA rheumatoid arthritis, Ab autoantibody, MTX-IR methotrexate inadequately responder, CD cluster designation

To assess the microvasculature at the synovial tissue level, each tissue was tested for the presence of CD31^+^ blood vessels. As shown in Fig. 4a–e, naïve PsA patients showed higher number of synovial CD31^+^ vessels (36.63 ± 11.02) compared to MTX-IR PsA patients (23.65 ± 11.67; *p* = 0.01). Similarly, naïve Ab^neg^ RA patients showed a higher number of synovial CD31^+^ vessels (35.43 ± 7.23) than MTX-IR Ab^neg^ RA patients (22.14 ± 5.32; *p* < 0.001). No significant differences in terms of CD31^+^ vessel count were found comparing PsA and Ab^neg^ RA patients stratified based on the same treatment category (Fig. 4e).

### Baseline synovial CD3^+^ and CD68^+^ cell IHC scores are associated with Minimal Disease Activity and remission achievement in PsA and Ab^neg^ RA patients naïve to treatment respectively

After study enrollment, each naïve patient started methotrexate treatment and was followed for at least 6 months on outpatient setting and treatment response rate was recorded at each clinical assessment (MDA for PsA and DAS remission for Ab^neg^ RA patients respectively). After 6 months follow-up from methotrexate beginning, 6 (50.0%) PsA patients achieved MDA, while 11 (40.7%) Ab^neg^ RA patients achieved DAS remission. As shown in Table 2, naïve PsA patients who reached the MDA status at 6 months follow-up had lower CD3^+^ cell IHC score (0.91 ± 0.33) before methotrexate beginning, compared to naïve PsA patients who did not achieve the clinical endpoint at 6 months (1.99 ± 0.84; *p* = 0.02). Conversely, naïve Ab^neg^ RA patients who reached the DAS remission status at 6 months follow-up had lower sublining CD68^+^ cell IHC score (0.92 ± 0.58) before methotrexate beginning, compared to naïve Ab^neg^ RA patients who did not achieved the clinical endpoint at 6 months (2.13 ± 0.55; *p* < 0.001) (Table 2). To define the best cut-off value for CD3^+^ cell scores and CD68^+^ cell sublining scores in naive PsA and Ab^neg^ RA respectively, ROC analysis was performed for each parameter (Additional file 1: Figure S1a-b). In particular, naive PsA patients who reached a MDA status after 6 months follow-up had more likely baseline CD3^+^ cell scores< 1.25 (83.3%) than naive PsA patients who did not reach MDA status (16.7%, *p* = 0.02). Moreover, naive Ab^neg^ RA patients who reached DAS remission after 6 months follow-up had more likely baseline CD68^+^ cell sublining scores < 2.25 (65.0%) than naive Ab^neg^ RA patients who did not reach DAS remission (0.0%, *p* = 0.03). In relation to MTX-IR patients, there was no significant difference in MDA and DAS remission status achievement in PsA and Ab^neg^ RA patients respectively, even stratifying patients based on the therapeutic strategy after MTX failure (c-DMARD combination or addition of b-DMARDs) (40.0% MTX-IR PsA patients achieved MDA after c-DMARD combination vs 64.7% MTX-IR PsA patients achieved MDA after b-DMARD addition, *p* = 0.32; 44.4% MTX-IR Ab^neg^ RA patients achieved MDA after c-DMARD combination vs 50.0% MTX-IR Ab^neg^ RA patients achieved MDA after b-DMARD addition, *p* = 0.78).

**Table 2:** IHC features of PsA and Ab^neg^ RA cohorts based on the achievement of MDA and DAS remission after 6 months follow-up

	PsA cohort					
	Naive (*N* = 12)		*p*	MTX-IR (*N* = 22)		*p* ^1^
	MDA (*N* = 6)	No MDA (*N* = 6)		MDA (*N* = 9)	No MDA (*N* = 13)	
Follicular synovitis, *n* (%)	3 (50.0)	3 (50.0)	1.00	3 (33.3)	7 (53.8)	0.34
CD68 (L) (mean ± SD)	1.58 ± 0.80	2.42 ± 0.73	0.09	1.86 ± 0.90	2.0 ± 1.07	0.77
CD68 (SL) (mean ± SD)	1.25 ± 1.08	2.16 ± 0.61	0.13	1.77 ± 0.93	2.0 ± 1.07	0.54
CD21 (mean ± SD)	0.58 ± 0.92	0.92 ± 1.02	0.59	0.96 ± 0.80	0.33 ± 0.43	0.06
CD20 (mean ± SD)	0.83 ± 0.82	1.17 ± 0.93	0.56	1.19 ± 0.83	1.06 ± 0.40	0.64
CD3 (mean ± SD)	*0.91 ± 0.33*	*1.99 ± 0.84*	*0.02*	1.65 ± 0.77	2.11 ± 0.96	0.29
CD117 (mean ± SD)	1.16 ± 0.82	1.42 ± 0.49	0.62	1.12 ± 0.62	1.39 ± 0.61	0.32
CD138 (mean ± SD)	0.67 ± 0.75	1.25 ± 0.88	0.25	0.58 ± 0.64	0.61 ± 0.60	0.84
CD31^+^ vessels (mean ± SD)	32.50 ± 13.41	40.75 ± 6.84	0.39	22.81 ± 11.31	25.21 ± 13.06	0.58
	Ab^neg^ RA cohort					
	Naive (*N* = 27)		*p* ^2^	MTX-IR (*N* = 28)		*p* ^3^
	REM (*N* = 11)	No REM (*N* = 16)		REM (*N* = 11)	No REM (*N* = 17)	
Follicular synovitis, *n* (%)	6 (46.2)	6 (42.6)	0.86	3 (27.3)	7 (41.2)	0.45
CD68 (L) (mean ± SD)	1.86 ± 0.82	2.23 ± 0.68	0.27	2.10 ± 0.51	1.97 ± 0.83	0.82
CD68 (SL) (mean ± SD)	*0.92 ± 0.58*	*2.13 ± 0.55*	*< 0.001*	1.35 ± 0.78	1.46 ± 0.76	0.66
CD21 (mean ± SD)	0.39 ± 0.74	0.65 ± 1.08	0.79	0.54 ± 0.79	0.77 ± 1.13	0.78
CD20 (mean ± SD)	1.64 ± 1.02	1.23 ± 0.78	0.26	1.46 ± 0.99	1.44 ± 0.83	0.85
CD3 (mean ± SD)	1.57 ± 0.95	1.57 ± 0.83	0.98	1.59 ± 0.89	1.47 ± 0.87	0.49
CD117 (mean ± SD)	0.57 ± 0.43	0.62 ± 0.46	0.79	0.77 ± 0.56	0.56 ± 0.43	0.28
CD138 (mean ± SD)	1.54 ± 0.99	1.77 ± 1.05	0.55	1.36 ± 1.07	1.62 ± 1.28	0.47
CD31^+^ vessels (mean ± SD)	35.64 ± 7.69	34.64 ± 6.89	0.54	22.35 ± 5.28	21.88 ± 5.78	0.52

Data presented in italics have *p* < 0.05

*PsA* psoriatic arthritis, *Ab* autoantibody, *RA* rheumatoid arthritis, *MDA* Minimal Disease Activity, *REM* remission, *DAS* Disease Activity Score, *p* naive PsA reaching MDA vs naive PsA not achieving MDA, *p*^*1*^ MTX-IR PsA reaching MDA vs MTX-IR PsA not achieving MDA, *p*^*2*^ naive AB^neg^ RA reaching DAS remission vs naive AB^neg^ RA not achieving DAS remission, *p*^*3*^ MTX-IR AB^neg^ RA reaching DAS remission vs MTX-IR AB^neg^ RA not achieving DAS remission, *MTX-IR* methotrexate inadequately responder

## Discussion

As in RA, synovial membrane inflammation plays a key pathogenetic role in PsA and many studies have focused on this topic in the last decades, especially looking for differential synovial tissue biomarkers between different types of chronic inflammatory joint diseases. More recently, the new advances in collecting synovial tissue through minimally invasive techniques have provided insight into the pathogenetic mechanisms of such joint diseases and facilitate differential diagnosis, stratification prognosis, and identification of treatment effects and new therapeutic targets [13].

In our study, we investigated, for the first time, the synovial histological features of a selected cohort of PsA patients, with peripheral joint involvement, compared to ACPA/RF seronegative RA cohort stratified based on the disease phase (disease onset and after DMARD insufficient response respectively) finding differential histological features of synovial tissue inflammation composition and biomarkers of therapeutic response.

Both RA and PsA are systemic autoimmune diseases characterized by chronic inflammation of the joint which leads to the destruction of the cartilage and bone [14, 15]. In particular, the clinical presentation of PsA is heterogeneous, variably involving the synovium of peripheral joints, spine, and/or entheses [6]. Moreover, PsA patients may develop articular structural damage both in terms of erosions and new bone formation and may also develop systemic complications including the development of metabolic syndrome and increased cardiovascular risk with concomitant reduced life expectancy [16, 17]. The synovium in PsA represents a primary target of disease pathogenesis, together with the skin and entheses, with a distinct gene signature compared to healthy and other joint diseases [18]. Thus, it is an intriguing and plausible site in exploring important mechanisms of the disease.

Multiple studies have characterized the histological features of synovitis in PsA compared with RA, proving that there are some substantial histological differences; notably, the PsA synovitis was shown to be characterized by less pronounced lining layer hyperplasia and fewer monocytes/macrophages than are seen in RA [19–21]. Given that it has already been clearly shown that oligoarticular and polyarticular PsA presents comparable histopathological characteristics [4], in PsA, there is a higher grade of synovial vascularization, with a different vascular pattern, characterized by immature, tortuous, and branched vessels, compared with the straight blood vessels more likely observed in RA [22].

Despite these advances, currently, there are no differential synovial tissue biomarkers between PsA and RA especially if the latter is diagnosed in a clinical setting of negativity for ACPA and RF antibodies. This particular setting may create difficulties in terms of differential diagnosis and prognosis in the earliest undifferentiated phase of the disease. To address this issue, we have previously demonstrated that synovial tissue analysis in terms of histological, ultrasound, and epigenetic signature may support the clinician in the identification of patients with ACPA/RF seronegative undifferentiated arthritis with high likelihood chance of clinical differentiation towards definite arthritis (PsA or RA) [23]. In particular, synovial tissue enriched with CD68^+^ and CD3^+^ cells and high CD31^+^ vessels characterized ACPA/RF seronegative undifferentiated arthritis patients evolving into Ab^neg^ RA or PsA [23].

Comparing PsA and Ab^neg^ RA, histological analysis of synovial tissue composition revealed similarities in lining and sublining CD68^+^ and sublining CD21^+^, CD20^+^, and CD3^+^ cell distribution. Previously, controversial data were reported about CD3^+^ cell distribution among PsA and RA. However, no study has been conducted selecting Ab^neg^ RA patients only as the comparison group and multiple methods of synovial tissue collection were used (arthroscopic or needle biopsies vs tissue obtained during joint replacement surgery) [24–26]. Other inflammatory cells such as mast cells and plasma cells take part in the tissue inflammatory infiltrate in PsA and RA [27, 28]. In particular, CD117^+^ cells have been previously shown more likely in synovial tissue of SpA, including PsA, expressing significantly more interleukin-17 than in RA synovitis regardless of TNF inhibition [27]. In our cohort, synovial tissue of PsA patients was found to be enriched in CD117^+^ cells in the sublining area compared to Ab^neg^ RA irrespective of the disease phase. Interestingly, CD117^+^ distribution was found more likely in the context of tissue lymphoid aggregates. Conversely, synovial tissue of Ab^neg^ RA patients was found to be enriched in CD138^+^ cells compared to PsA synovial tissue, underlining the crucial role of B lymphocytes in RA pathogenesis and suggesting the need to investigate additional autoantibody specificities despite ACPA/RF negativity in such patient category. Moreover, the detection of lympho-neogenesis is a frequent feature of PsA synovitis with the expression of peripheral lymph node addressin-positive high endothelial venules and CXCL13/CCL21 expression demonstrating that the microanatomical bases for germinal center formation are present in PsA synovial tissue [29]. In this context, the concept of the autoimmune nature of PsA disease is strengthened by the recent detection of autoantibodies against modified antigens in the peripheral blood and synovial tissue of early naive to treatment PsA patients [30].

Increased vascularity has been reported in both psoriatic skin lesions and synovial tissue in PsA. In the dermis of the psoriatic skin, an abundance of dilated and tortuous blood vessels is present [31]. Multiple authors have reported that PsA synovium is characterized by an increase in macroscopically tortuous blood vessels, and this is more pronounced in, but not exclusive to, PsA than it is in RA synovium [20, 32]. In our study cohort, CD31 IHC revealed that the mean number of synovial CD31^+^ vessels does not differ among PsA and Ab^neg^ RA, suggesting that the microscopical level does not mirror the macroscopical view of synovial vasculature in PsA compared to RA once disease phase stratification is done.

The development of novel biomarkers of therapeutic response prediction is urgently needed for PsA and RA management. Previous studies have investigated the effect of histological markers of synovitis in PsA and RA mirroring the therapeutic response to conventional and biological DMARDs respectively [33–35]. In particular, Pontifex et al. proposed IHC score for sublining CD3^+^ cells as a useful biomarker of treatment response in PsA, enrolling a PsA cohort treated with TNF inhibitor [36] without providing clear cut-off value to be used at treatment initiation in naive PsA patients. In our study cohorts, we found that naive to treatment PsA with high likelihood of MDA achievement after DMARD treatment was more likely characterized, at baseline, by IHC score for CD3^+^ cells < 1.25 compared to PsA patients not achieving this clinical outcome. Conversely, validation studies in RA patients, regardless of the autoimmune profile, found that variation in IHC score of sublining CD68^+^ cells is a valuable synovial biomarker mirroring the treatment response to conventional and biological DMARDs (rituximab and TNF inhibitor) [37–39]. Interestingly, in our study, we confirmed these findings in a well-selected naive to treatment Ab^neg^ RA patient cohort in which Ab^neg^ RA patients with high likelihood chance of DAS remission achievement after DMARD treatment were more likely characterized, at baseline, by IHC score for sublining CD68^+^ cells < 2.5 compared to Ab^neg^ RA patients not achieving DAS remission after DMARD treatment.

## Conclusions

In conclusion, our comparative study assessing the histological features of synovial tissues obtained from naive to treatment and MTX-IR PsA and Ab^neg^ RA patients revealed that PsA synovitis is characterized by being mast cell (CD117^+^) rich but plasma cell (CD138^+^) poor whereas Ab^neg^ RA synovitis by the reverse findings, being plasma cell (CD38^+^) rich but mast cell (CD117^+^) poor. These different histopathologic biomarkers may help to solve the diagnostic overlapping issue in the setting of ACPA/RF negativity at the disease onset. Moreover, baseline IHC scores of CD3^+^ and sublining CD68^+^ arose as useful biomarkers of treatment response to first-line DMARDs in PsA and Ab^neg^ RA respectively, suggesting the need to include both parameters in more extensive future synovial tissue biopsy-driven clinical trials for these inflammatory joint conditions.

## Additional files

Additional file 1: **Figure S1.** (a-b) ROC curve analysis for cut-off values for CD3^+^ cells and SL CD68^+^ cells IHC in naive PsA and Ab^neg^ RA patients. SL sublining, PsA psoriatic arthritis, Ab autoantibody, RA rheumatoid arthritis, IHC immunohistochemistry. (TIF 12416 kb)

Additional file 2: **Table S1.** Inter-rater agreement coefficients for CD68, CD21, CD20, CD3, CD117, CD138, and CD31 IHC scores. (DOCX 12 kb)

## Abbreviations

Ab^neg^: Autoantibody negative
ACPA: Anti-citrullinated peptide antibody
CD: Cluster designation
CRP: C-reactive protein
DAPSA: Disease Activity in PSoriatic Arthritis
DAS: Disease Activity Score
ESR: Erythrocyte sedimentation rate
IHC: Immunohistochemistry
MDA: Minimal Disease Activity
MTX-IR: Methotrexate inadequately responder
PsA: Psoriatic arthritis
RA: Rheumatoid arthritis
REM: Remission
RF: Rheumatoid factor
SJC: Swollen joint count
TJC: Tender joint count
