# Supplementary figures and images for: Differential synovial tissue biomarkers among psoriatic arthritis and rheumatoid factor/anti-citrulline antibody-negative rheumatoid arthritis
Source: Arthritis Res Ther. 2019 May 9;21:116. doi: 10.1186/s13075-019-1898-7 (PMC6509792; doi:10.1186/s13075-019-1898-7)

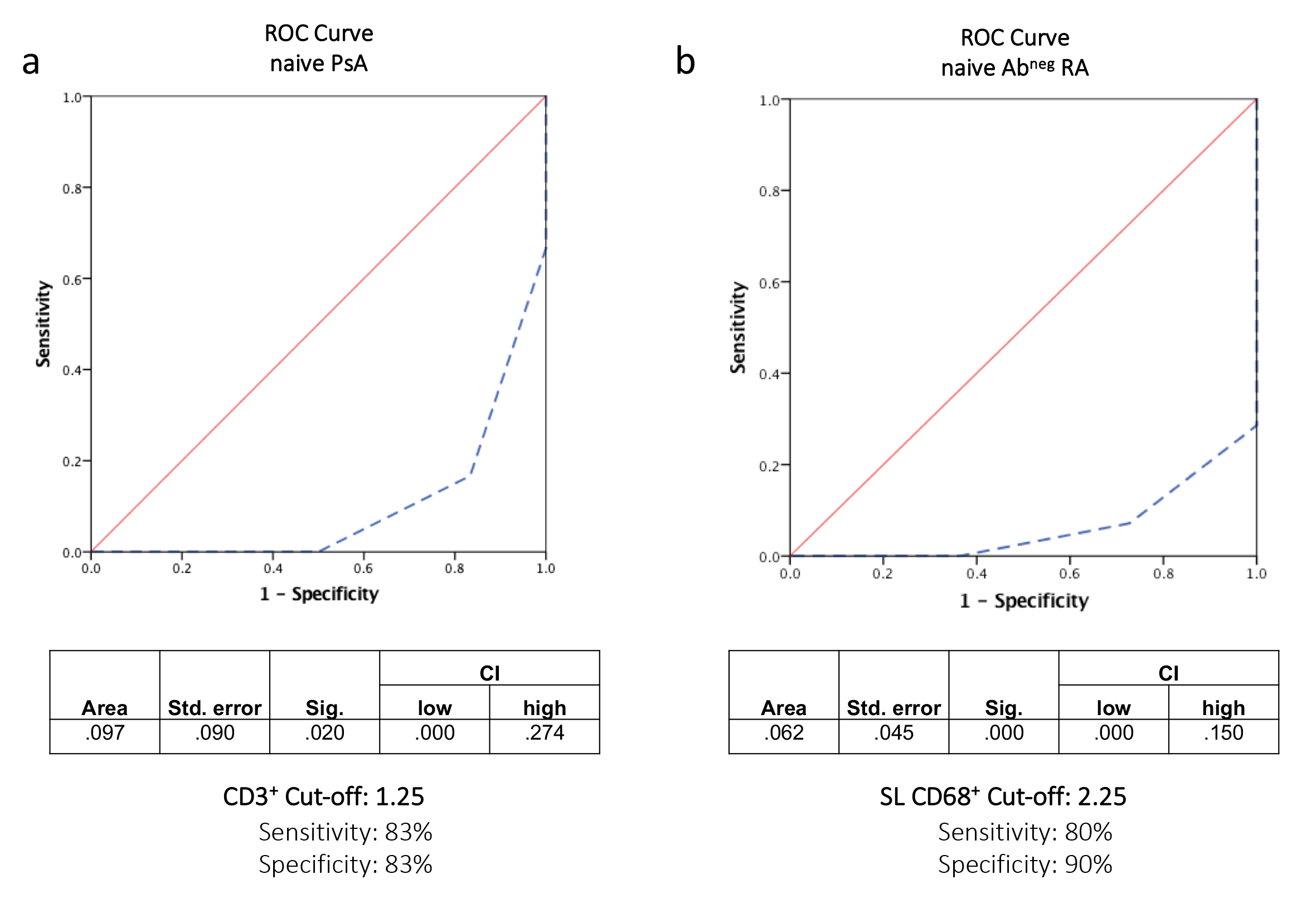

Supplement: Supplementary file 1 — Figure S1. (a-b) ROC curve analysis for cut-off values for CD3+ cells and SL CD68+ cells IHC in naive PsA and Abneg RA patients. SL sublining, PsA psoriatic arthritis, Ab autoantibody, RA rheumatoid arthritis, IHC immunohistochemistry. (TIF 12416 kb) [file 13075_2019_1898_MOESM1_ESM.tif]
