# Supplementary material for: Differential synovial tissue biomarkers among psoriatic arthritis and rheumatoid factor/anti-citrulline antibody-negative rheumatoid arthritis
Source: Arthritis Res Ther. 2019 May 9;21:116. doi: 10.1186/s13075-019-1898-7 (PMC6509792; doi:10.1186/s13075-019-1898-7)
Supplement: Supplementary file 2 — Table S1. Inter-rater agreement coefficients for CD68, CD21, CD20, CD3, CD117, CD138, and CD31 IHC scores. (DOCX 12 kb) [file 13075_2019_1898_MOESM2_ESM.docx]

**Table S1.** Inter-rater agreement coefficients for CD68, CD21, CD20, CD3, CD117, CD138 and CD31 IHC scores.

|  | **Inter-rater coefficient*** |
| --- | --- |
| **(L) CD68 IHC score** | R=0.585; p<0.001 |
| **(SL) CD68 IHC score** | R=0.730; p<0.001 |
| **CD21 IHC score** | R=0.791; p<0.001 |
| **CD20 IHC score** | R=0.745; p<0.001 |
| **CD3 IHC score** | R=0.810; p<0.001 |
| **CD117 IHC score** | R=0.594; p<0.001 |
| **CD138 IHC score** | R=0.735; p<0.001 |
| **CD31 synovial vessels count** | R=0.876; p<0.001 |

**IHC**: Immunohistochemistry; **(L)** Lining; **(SL)** Sublining; *Pearson Correlation coefficient.
